# The Function of the PRRSV–Host Interactions and Their Effects on Viral Replication and Propagation in Antiviral Strategies

**DOI:** 10.3390/vaccines9040364

**Published:** 2021-04-09

**Authors:** Jun Ma, Lulu Ma, Meiting Yang, Wei Wu, Wenhai Feng, Zhongzhou Chen

**Affiliations:** State Key Laboratory of Agrobiotechnology and Beijing Advanced Innovation Center for Food Nutrition and Human Health, College of Biological Sciences, China Agricultural University, Beijing 100193, China; majunjun@cau.edu.cn (J.M.); mll0703@cau.edu.cn (L.M.); meitingyang@cau.edu.cn (M.Y.); wuweiyou@cau.edu.cn (W.W.); whfeng@cau.edu.cn (W.F.)

**Keywords:** PRRSV, host, interaction, immune responses, vaccines

## Abstract

Porcine reproductive and respiratory syndrome virus (PRRSV) affects the global swine industry and causes disastrous economic losses each year. The genome of PRRSV is an enveloped single-stranded positive-sense RNA of approximately 15 kb. The PRRSV replicates primarily in alveolar macrophages of pig lungs and lymphatic organs and causes reproductive problems in sows and respiratory symptoms in piglets. To date, studies on how PRRSV survives in the host, the host immune response against viral infections, and pathogenesis, have been reported. PRRSV vaccines have been developed, including inactive virus, modified live virus, attenuated live vaccine, DNA vaccine, and immune adjuvant vaccines. However, there are certain problems with the durability and effectiveness of the licensed vaccines. Moreover, the high variability and fast-evolving populations of this RNA virus challenge the design of PRRSV vaccines, and thus effective vaccines against PRRSV have not been developed successfully. As is well known, viruses interact with the host to escape the host’s immune response and then replicate and propagate in the host, which is the key to virus survival. Here, we review the complex network and the mechanism of PRRSV–host interactions in the processes of virus infection. It is critical to develop novel antiviral strategies against PRRSV by studying these host–virus interactions and structures to better understand the molecular mechanisms of PRRSV immune escape.

## 1. Introduction

Porcine reproductive and respiratory syndrome virus (PRRSV) has undoubtedly become a substantial financial issue that has affected pig production and caused substantial economic losses for the swine industry worldwide since its outbreak in the 1980s [1,2]. Annual economic losses in the US due to PRRSV manifested in reproductive problems in sows and respiratory symptoms in piglets were assessed as reaching USD 664 million in 2013 [3,4]. The latest economic estimate in Germany indicated the losses on farm profits due to the PRRS virus were −19.1% on average and −41% in the worst case [2]. As a global swine pathogen that has caused catastrophic economic losses, PRRSV is the cause for continuing and widespread concern [5].

As a member of the Nidovirales order in the Arteriviridae family, PRRSV is an enveloped virus with an average diameter of 55 nm; it is a positive-stranded RNA virus with an approximately 15 kb genome with a 5′ cap and a 3′ poly A tail [4,6]. Except for the 5′ and 3′ untranslated regions at both ends, the PRRSV genome contains at least 11 known open reading frames (ORFs) [7]. The first two ORFs occupy about 75% of the viral genomes coding for polyproteins, including pp1a and pp1ab, by ribosome shifting, and then the PRRSV proteases hydrolyze and cleave polyproteins into at least 16 distinct nonstructural proteins (nsps) [4,8]. The nsps that participate in viral genome replication and transcription are essential for the survival of the PRRSV [9]. PRRSV virions are composed of an N protein (nucleocapsid protein) and a lipid envelop (GP2, E, GP3, GP4, M, GP5, ORF5a) that envelops the membrane [10,11]. M and GP5 are the major components of the virus coat [12].

The PRRSV is mainly divided into two genotypes: type 1 (represented by the European strain Lelystad Virus) and type 2 (represented by the North American strain VR-2332), and both PRRSV genotypes have only 50–60% nucleotide identity [6,13]. In addition to the genotype differences between PRRSV-1 and PRRSV-2, the host immune responses have been shown to differ, sometimes considerably due to their biological differences including pathogenicity (Table 1). A large number of studies have found a general consensus that PRRSV-2 causes more severe respiratory disease than PRRSV-1 [14]. Therefore, it is necessary to emphasize the host immune response induced by different genotypes. Due to high inter-strain genetic exchange and rapid mutations of PRRSV, it has always been a substantial challenge to design effective vaccines and drugs [4].

## 2. The Process of the PRRSV Entry and Infection

**Figure 1:**
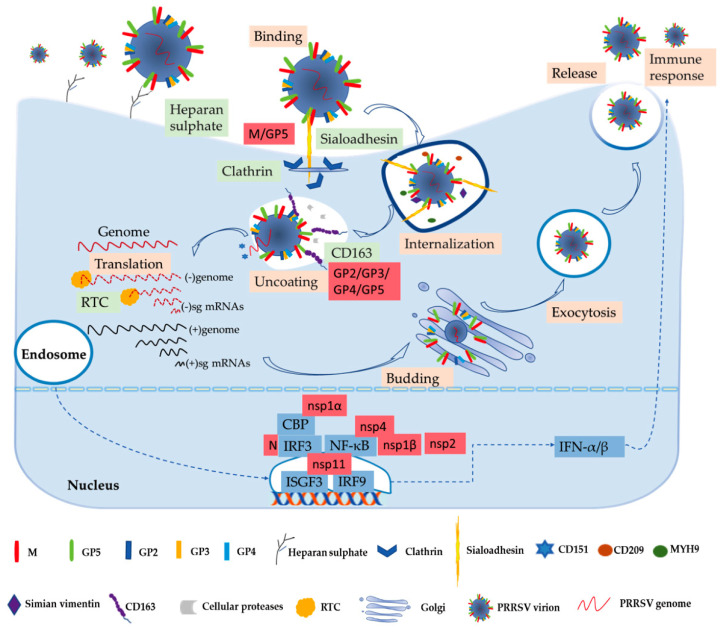
PRRSV replication and infection cycle, including binding, internalization, uncoating, translation, assembling, releasing the virion, and immune response. The cytokine IFN-α/β stimulates host innate and adaptive immune response against PRRSV infection first. In this process, some important viral structural and non-structural proteins interact with host factors.

Numerous studies have been performed to learn more about the biochemistry of the PRRSV. The process of the virus entry into its host cell is the first crucial step in the infection and the focus of basic research [53]. PRRSV has a very narrow cell tropism, and the primary target cells are porcine alveolar macrophages (PAMs) [12,54]. Numerous studies have found that the existence of the specific entry mediators in the target cell leads to its restricted cell tropism [53,55]. These cellular factors, including heparan sulphate, CD163, porcine sialoadhesin (pSn), vimentin, CD151, CD209 (DC-SIGN), non-muscle myosin heavy chain 9 (MYH9), and others, are involved in virus binding, internalization, and genome release, as shown in Figure 1 [53,56].

In the early stages, PRRSV enters the macrophage via a receptor-mediated method [53,57]. PRRSV concentrates virions on the cell’s surface by interacting with heparan sulphate glycosaminoglycans that are present on the surface of mammalian cells, hence promoting a more efficient attachment for subsequent binding to one or more receptors involved in virus internalization; studies have found that PRRSV-1 and PRRSV-2 have different sensitivities to heparin [15,53,58]. Next, the integration with pSn gradually increases. The pSn, a PAMs-restricted type 1 transmembrane glycoprotein, is identified as a PRRSV attachment and internalization receptor via clathrin-mediated endocytosis [53,58]. The heparan sulphate enhances the interaction between the virus and sialoadhesin, but is not necessary, and the M/GP5 complex is involved in interacting with heparan and the N-terminal domain of pSn [15,16]. Upon internalization with the participation of pSn, the genome of the virus that is present in the early endosome is released into the cytoplasm [53]. The last stage is critically dependent on CD163, a scavenger receptor cysteine-rich (SRCR) family for hemoglobin clearance, which is the most specific and indispensable receptor for PRRSV entry and infection both in vitro and in vivo [8,12,59]. Additionally, the viral GP2 and GP4 glycoproteins bind to CD163 [13]. Moreover, a study has shown that CD163 interacts with GP3 and GP5 in addition to the known interactions with GP4 and GP2, and co-immunoprecipitation (co-IP) analysis indicated that the SRCR5-domain deletion of CD163 loses its interaction with viral GP2, GP3, and GP5, and thus blocks virus uncoating in the early endosomes [17]. A growing body of research has also indicated that CD163 plays an essential role in viral uncoating and genome release, and CD163 knockout pigs are resistant to PRRSV infection [13,59,60,61]. In addition to CD163, cellular proteases, such as the aspartic protease cathepsin E and trypsin-like serine proteases, have been implicated in the uncoating process and subsequent infection [62]. Moreover, there are some mediators involved in the entry process of PRRSV into the macrophage. CD151 interacting with the PRRSV-2 3′ untranslated region (UTR) RNA should cooperate in infection in vitro [18]. Simian vimentin bound to PRRSV-2 nucleocapsid protein is involved in mediating transportation of the virus in the cytosol [19]. CD209 (DC-SIGN) and MYH9 interacting with GP5 are essential factors in both PRRSV-1 and PRRSV-2 entry and infection [20,21,22].

The genome released by the virus was identified as the template used to translate into pp1a and pp1ab, and after that the proteases hydrolyze and cleave polyproteins into mature nonstructural proteins as described above [63]. The viral replication and transcription complex (RTC) is assembled by nsps, whose key components are nsp9 and nsp10 [64]. The RTC first engages in producing both full-length and subgenome (sg)-length minus strands using a mechanism of discontinuous transcription, the latter serving as templates for the synthesis of plus-strand sg mRNAs required to express the structural protein genes, which reside in the 3′-proximal quarter of the genome [8,65,66]. The last stage is assembling and releasing the virion. Novel generated RNA genomes are packaged into nucleocapsids that become enveloped virions by structural proteins through budding from smooth intracellular membranes, and the new virions are released from the cell in the form of exocytosis [8,65]. The entire process is illustrated in Figure 1.

## 3. The PRRSV–Host Interactions

The complex networks of virus–host interactions are essential in the overall process of PRRSV entry and infection. The viral structural proteins interact with host receptors to mediate viral entry as described above. The interactions of host and viral nonstructural proteins can exert an influence on the replication and transcription of viral genomes. The virus’s invasion causes a series of immune responses, and then virions escape from the host immune system to favor their own replication by interacting with the host. Only by better understanding the molecular mechanism of PRRSV immune evasion and modulation can we design more effective vaccines. Further studies of these host–virus interactions are critical to the development of novel antiviral strategies against PRRSV.

### 3.1. Interferons (IFNs)

Innate immunity is the first line of defense without memory and specificity, and interferons (IFNs) are one of the earliest cytokines and major elements of the host fighting with virus invasion, as shown in Figure 1 [13,67]. PRRSV-2 is sensitive to type I interferons (IFN-α/β), and has some immunosuppressive mechanisms of suppressing IFNs [13,68]. Activation of IFN regulatory factor 3 (IRF3) and nuclear factor-κB (NF-κB) plays an important role in activating the IFN-β promoter, and these factors bind to the IFN-β promoter to form an enhanceosome via the cAMP response element-binding (CREB)-binding protein (CBP) transcriptional co-activator [23]. This also suggests that the virus has evolved to assuage the host’s innate immunity [13]. The mechanism of suppressing IFNs by blocking the activation of the IRF3 or NF-κB is an important strategy to respond to the innate immunity of the host to the virus’s proteins, such as nsp1, nsp2, and nsp4 [27,28]. Some studies have revealed that IFNs are suppressed by the PRRSV-2′s nsp1, which is a potent IFN antagonist [24]. The zinc finger 1 motif of nsp1α is essential in suppressing IFNs by inducing CBP degradation to inhibit the recruitment of CBP for enhanceosome assembly, which is likely the key mechanism in IFN suppression [23,24]. Moreover, nsp1β inhibits both IRF3- and NF-κB-dependent gene induction via dsRNA and the Sendai virus, resulting in IFN suppression [25]. Similarly, the PRRSV-2 nucleocapsid (N) protein inhibits the phosphorylation and nuclear translocation of IRF3 to suppress IFNβ induction [26]. Although nsp11 endoribonuclease (NendoU) activity inhibits IFNβ by suppressing IRF3 and NF-κB activation, binding to IRF9 controls the formation and nuclear translocation of the IFN-stimulated gene factor 3 (ISGF3) and antagonizes type I IFN signaling in a NendoU activity-independent manner [29,30,31].

### 3.2. Interleukin (IL)

In highly pathogenic (HP) PRRSV-infected swine, the cytokines including interleukin (IL)-1, IL-6, IL-8, IL-10, and tumor necrosis factor (TNF)-α are significantly increased [69,70]. The PRRSV-2 envelope protein E remarkably increases the release of IL-1β from lipopolysaccharide (LPS)-primed PAMs [32]. Studies showed that nsp11 NendoU activity plays a key role in inhibiting the secretion of IL-1β [33]. IL-10 is a significant immunosuppression cytokine with anti-inflammatory properties that can counteract adaptive immunity, thereby preventing impairment to the host [71,72]. A previous study has demonstrated that the PRRSV-2 N protein can up-regulate IL-10 via NF-κB and p38 mitogen-activated protein kinase (MAPK) pathways in PAMs [73]. Additionally, in porcine monocyte-derived dendritic cells (MoDCs) and PAMs, the N protein can promote the expression of IL-10 [34]. The GP5 could significantly increase IL-10 production through p38 MAPK and signal transducer and activator of transcription-3 (STAT3) activation [36]. Similarly, nsp1 can increase the level of IL-10 as an inducer in vivo [35]. IL-17 is a proinflammatory cytokine associated with intense inflammation and is upregulated by HP-PRRSV of genotype 2 infection [13,37]. A study found that HP-PRRSV nsp11 could induce IL-17 production depending on the phosphatidylinositol 3-kinase (PI3K)-p38MAPK-C/EBPβ/CREB pathways [37].

### 3.3. Tripartite Motif (TRIM) Proteins

Tripartite motif (TRIM) proteins, as critical components of the innate immune system, play significant roles in fighting virus invasion for mammalian cells, and can regulate multiple cellular processes including transcription-dependent antiviral responses such as cytokine-mediated or autophagy-mediated antiviral modulation [40,74,75]. The N-terminal RING-type zinc finger domain of TRIM proteins confers E3 ubiquitin ligase activity [74]. Study has indicated that the PRRSV-2 N protein can antagonize the antiviral activity of TRIM25 and suppress innate immune responses of the host by competitively interacting with TRIM25, thereby interfering with TRIM25-mediated retinoic acid-inducible gene I (RIG-I) ubiquitination and inhibiting IFN-β production [38]. TRIM22 can interact with the PRRSV-2 N protein and reduce virus replication, and the SPla and the RYanodine Receptor (SPRY) domain and nuclear localization signal of TRIM22 are indispensable for this interaction [39]. Moreover, the N-terminal RING domain of TRIM59, which is an important antiviral component, can interact with the C-terminal NendoU domain of nsp11, thereby inhibiting PRRSV-2 infection [40].

### 3.4. MicroRNAs (miRNAs)

MicroRNAs (miRNAs) are small non-coding RNA molecules containing about 21 nucleotides in length, and they play key roles in the complex networks of PRRSV–host interactions [76,77]. More recently, miRNAs have been considered as crucial post-transcriptional gene regulators for viral replication and host immune responses in the process of PRRSV invasion [13,77,78]. This research had indicated that MicroRNA can target signaling pathways or host factors both related to PRRSV replication [5]. MiR-30c has a negative effect on the IFN-I response by targeting Janus kinase 1 (JAK1) to facilitate HP-PRRSV of genotype 2 infection [79]. On the contrary, miR-181 can suppress PRRSV-2 infection by targeting and down-regulating the PRRSV-2 receptor CD163 [41]. MiR-10a-5p can interact with 3′ UTR of pig SRP14 mRNA and reduce SRP14 expression through translational repression, thereby inhibiting PRRSV-1 replication [80]. Additionally, miRNAs also can target the PRRSV genome directly, and this is a new perspective on controlling PRRSV infection [81]. In addition to targeting the 3′ UTR of CD163 mRNA, MiR-181 can combine with ORF4, which is a highly conserved region of PRRSV-2 genomic RNA, and thus inhibits PRRSV-2 replication [41,42]. MiR-130 can directly target the HP-PRRSV 5′ UTR and exert antiviral activity. Further study has reported that miR-130 has an effect on inhibiting the replication of PRRSV-2 strains, but not the replication of classical PRRSV-1 strains [13,43]. MiR-23 and miR-378, which were identified as critical inhibitors of PRRSV-2 replication, can directly target PRRSV genomic ORF3 and ORF7, respectively [44]. Interestingly, miR-505, also as a critical inhibitor of PRRSV-2 replication, can directly target both ORF3 and ORF5.

### 3.5. Other Host Factors’ Interactions with PRRSV

PRRSV depends on host factors to complete genome replication and interacts with host molecules for its survival and reproduction. The host immune system will take some measures to suppress the virus replication during virus invasion. Besides the abovementioned host factors, other cellular components are also involved in interacting with PRRSV. The cellular protein nucleoporin 62 (Nup62) interacts with nsp1β, leading to inhibition of host antiviral protein expression, revealing a new strategy of immune escape [47]. In addition, research found that cellular retinoblastoma protein (pRb) interacts with the nsp9 of genotype 2 PRRSV, which will benefit the replication of PRRSV-2 [49]. Inversely, the leucine-rich repeats (LRR) domain of nucleotide-binding oligomerization domain-like receptor (NLR) X1 as a new host restriction factor interacts with the RdRp domain of PRRSV-2 nsp9, which restricts PRRSV-2 replication [50]. Similarly, the interaction of the zinc finger domain of ZAP, a zinc finger antiviral protein, and nsp9 mapped to amino acids 150 to 160, can repress PRRSV-2 replication [51]. Nsp1α can combine with the protein inhibitor of activated STAT1 (PIAS1) as the small ubiquitin-related modifier (SUMO) E3 ligase leading to the nsp1α sumoylation [45]. In virtue of the SUMO E3 ligase activity, nsp1α interacts with swine leukocyte antigen class I (SLA-I) to modulate degradation, in the same way facilitating the ubiquitinylation of CBP for degradation [45,46]. The nsp5 of PRRSV-1 and PRRSV-2 was shown to promote the degradation of signal transducer and activator of transcription 3 (STAT3), a pleiotropic signaling mediator of numerous cytokines, leading to interference with the JAK/STAT3 signaling and the host immune responses [48]. The PRRSV-2 N protein interacts with RNA-associated nuclear host proteins such as fibrillarin, nucleolin, and poly(A)-binding protein, but the specific function remains to be further clarified [52]. Furthermore, the PRRSV N protein appears to up-regulate NF-κB activation that is attributed to the N protein nuclear localization signal [45].

In conclusion, a complex network of PRRSV–host interactions exists throughout the virus cycle, including virus entry, replication, and infection, as shown in Table 1. The immune response caused by different virus genotypes is emphasized in Table 1. Although significant advancements have been made, the understanding of direct or indirect virus–host interaction networks remain limited. Moreover, some seemingly contradictory results are hard to explain. Study indicated that the NF-κB is stimulated by the N protein, which may up-regulate IFN [45]. However, the N protein inhibits the phosphorylation and nuclear translocation of IRF3 to suppress IFNβ induction [26]. The stimulation of NF-κB by the N protein may involve other cellular pathways, and the mechanism of cytokines’ regulation by the N protein needs to be further explored. The interaction of PRRSV nsp9, a critical component of the viral RTC, and some host proteins can inhibit virus replications such as NLRX1 and ZAP, and yet they can promote virus replications such as pRb, which has aroused much concern.

## 4. Current Antiviral Strategies and Prospects

Since the outbreak of PRRSV in the 1980s, efforts have been made vehemently to find effective antiviral strategies and vaccines to control its damage to the swine industry. However, for a variety of reasons, this has become a huge challenge. Modified-live virus (MLV) vaccines and inactivated vaccines against PRRSV are available and used worldwide [56]. Both non-structural and structural proteins are targets of PRRSV modified attenuation [82]. It was found that nsp2, nsp3, nsp10, GP2 and GP5 are major proteins involved in virus attenuation [82]. Particularly, nsp2 and GP5 are the most variable PRRSV proteins, and mutations generally occurred in the hypervariable regions of these two proteins [83]. For instance, HP-PRRSV MLV TJM-F92 has a unique 120-amino acid deletion located in the 628–747 residues of nsp2 [83,84]. The CH-1R MLV vaccines against classical PRRSV strains were developed from the North American strains CH-1a [85]. A total of 54 mutated amino acids were found in CH-1R; among them, 11 and three mutations are located in nsp2 and GP5, respectively [82]. PRRS-MLV can protect homologous viruses and mitigate lung damage but does not induce sufficient heterologous immunity [86]. Compared with PRRSV-1 MLV vaccines, PRRSV-2 MLV vaccines are more effective against both PRRSV-1 and PRRSV-2 infection [87,88]. In addition to the issue of immunity breadth to fight new outbreaks, MLV may revert to virulence, and safety issues have aroused concerns [89]. The safety of inactivated vaccines is sufficient, but their immunogenicity is poor [90]. The combination of immune adjuvant and inactivated vaccines can enhance vaccine efficacy [89]. It has been reported that intranasal delivery of a poly(lactic-co-glycolic acid) (PLGA) nanoparticle (NP)-entrapped inactivated PRRSV vaccine has the potential to induce a broadly cross-protective immune response [90]. In addition, the intranasal Th1-biased adjuvant of the recombinant B subunit of the Escherichia coli heat-labile enterotoxin rLTB can significantly strengthen cellular immune responses [89]. Whether subunit vaccines, DNA vaccines or virus vectored vaccines can replace MLV remains to be further studied [56]. Moreover, the rapid mutation of this RNA virus, which leads to a high degree of genetic and antigenic variation, challenges the design of PRRSV vaccine. Thus, there is an urgent need to improve upon the existing vaccines, which mainly refers to the enhancement of the breadth of immunity and creating cross-protective virus vaccines [91]. PRRSV nsp4- or nsp9-specific nanobodies can inhibit PRRSV replication by blocking these nsps, which are critical components of the viral RTC, therefore interfering with viral genome replication and transcription [92,93]. There are some relatively conserved nonstructural proteins, such as nsp1, nsp4, nsp9, and nsp11, that play important roles in the life cycle of viral infection according to our summary of the PRRSV–host interaction network, and nanobodies/inhibitors that target these proteins offer a novel perspective on antiviral strategies that may potentially resist PRRSV infection across genotypes. According to protein data bank (PDB) information, the structures of nsp1α (PDB code: 3IFU), nsp1β (PDB code: 3MTV), nsp10 (PDB code: 6JDU), and nsp11 (PDB code: 5EYI), provide structural basics for the study of biological function and action mechanism [94,95,96,97,98]. For example, the C-terminal extension of nsp1β binds to the putative substrate binding site of the papain-like cysteine protease domain, which illustrates the role of the substrate binding mode and provides a structural template to design nsp1 inhibitors with potential therapeutic value [96].

Classically attenuated viruses are created by passaging viruses in cultured cells and are effective for many viruses. However, many safety issues exist for these empiric attenuations [99]. Furthermore, one major drawback of the current live-attenuated PRRSV vaccines is that they cannot distinguish PRRSV-specific antibodies produced by natural infection with wild type (WT) viruses or by vaccination [100]. Therefore, a differentiating infected from vaccinated animals (DIVA) vaccine is extremely useful to survey and eradicate PRRSV, and has become an important aim of many current research efforts [101]. Epitope-M201, which is highly immunodominant and well-conserved among PRRSV-2 isolates, is located at the carboxyl terminus (residues 161–174) of the viral M protein, and provides a serologic marker for the development of live-attenuated DIVA vaccines against PRRSV-2 [100]. A2MC2-P90, which has a 181 residue deletion in nsp2 and 35 nucleotide mutations throughout the genome, could serve as an ideal and significant backbone for DIVA vaccine development due to its unique properties including the ability to induce IFN, a virulence in swine and its largest spontaneous deletion in nsp2 [56,102]. Advances in viral structures have provided new ways of controlling viral replication and virulence. These MLV vaccines may lead to a new generation of safer, more widely applicable PRRSV vaccines. As shown in Table 1, PRRSV structural envelope proteins play vital roles in the viral infection cycle. Unfortunately, the structures of these proteins are not available, making it difficult to understand the structural basis and molecular mechanisms of the virus–receptor interaction or antibody-mediated neutralization, so as to guide the optimization of vaccines and to improve immunogenicity [103]. However, a structure-based vaccine design approach can be used to improve the antigenicity and to create immunogens capable of eliciting robust neutralizing and protective immune responses at the atomic level. Furthermore, structure-based specific MLV design can be used to generate many kinds of MLVs without the risk of reverting to virulence.

Many viral proteins, such as nsp1α, nsp1β, nsp2, nsp4, nsp7, nsp10, and nsp11, play an essential role in suppressing the host innate immune response. These key proteins can be used as antiviral targets for attenuated vaccines. For example, the nsp4 D185N mutant exhibits a slower replication rate and a higher ability to induce IFN-I expression compared with WT PRRSV [104]. Additionally, based on the 3D structure of nsp2, mutated viruses lacking deubiquitinating enzyme activity exhibit WT replication kinetics, but strikingly enhance innate immune signaling [105]. The IFN-β mRNA levels are increased more than 10-fold compared with those in the WT virus-infected cells. These modified viruses display a significantly weakened ability to evade host immune responses, opening new possibilities for developing improved attenuated virus vaccines against economically important arteriviruses. Moreover, PRRSV nsp11 inhibits NF-κB signaling using its deubiquitinating activity [33]. Thus, selective mutations of the conserved sites C112 and Y219 of deubiquitinating activity will greatly increase host innate immune responses without affecting endoribonuclease activity. These mutations can be effectively applied to live attenuated PRRSV vaccines, thus providing the basis for a DIVA vaccine. Moreover, the combination of specific MLVs and immune adjuvants can further enhance vaccine efficacy.

Furthermore, the identification of miRNAs that target the PRRSV genome provides alternative targets for gene editing [5]. With the rapid development of CRISPR/Cas-based gene editing technology, the acquisition of gene-edited pigs is no longer a difficult task. In this context, the TRIM22 gene has been lost during evolution, and restoring TRIM22 through gene editing will be a potential antiviral strategy [39]. Furthermore, gene-editing of CD163, which has been determined to be the major receptor, generates pigs resisting PRRSV infection [61]. Notably, the crystal structure of the SRCR5 domain of CD163 (PDB code: 5JFB) consists of seven β-strands and two α-helices, which extends our understanding of the mechanism of PRRSV invasion [103]. Arg561, which is located in the long loop region, is an important residue for PRRSV invasion and may play a key role in the interaction between CD163 and PRRSV during viral infection, which provides a target for drug design and gene editing. However, the acceptance of genetically modified organisms as food challenges the development of these antiviral strategies, and thus biosafety should be increasingly emphasized in the development of new virus vaccines.

**Figure 2:**
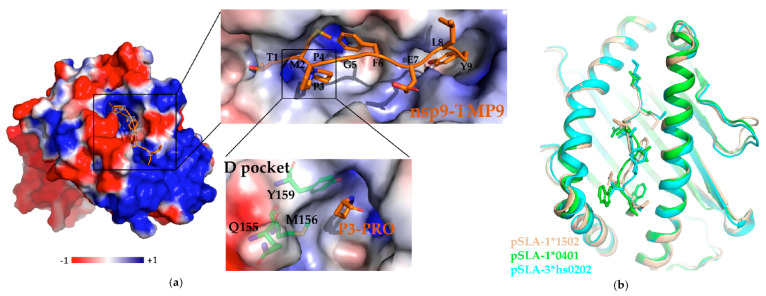
D pocket structure of pSLA-1*1502 bound to nsp9-TMP9 peptide, and comparison of the peptide conformations of the three pSLA structures resolved. (**a**) Overall structure of pSLA-1*1502 complexes with one peptide (nsp9–TMP9) (Protein Data Bank (PDB) code: 5YLX) is represented by electrostatic surface (red, negatively charged; white, non-polar; and blue, positively charged). The nsp9–TMP9 is shown as a stick model (C, orange; N, blue; O, red). The D pocket is shown in particular; P3-PRO represents the third proline residue of the nsp9–TMP9 peptide, and Q155, M156, Y159 residues shown as stick models (C, green; N, blue; O, red) in complexes can interact with the P3-PRO by van der Waals forces. (**b**) The superposition of the pocket structures of pSLA-1*1502 (wheat, PDB code: 5YLX), pSLA-1*0401 (green, PDB code: 3QQ3), and pSLA-3*hs0202 (cyan, PDB code: 5H94) are shown as cartoons. There are slight differences in the conformation of peptides shown as stick models in the three structures.

Subunit peptide vaccines reproducing the B and T epitopes are a novel antivirus strategy [106]. Cytotoxic T lymphocytes (CTLs) epitope vaccines have superiorities in terms of specificity, safety and clinical effect, and have been successfully used to fight viral infections, such as human immunodeficiency virus (HIV), human papilloma virus (HPV), and dengue virus [107,108,109]. The presentation of viral epitopes to CTLs by SLA-1 plays a key role in swine immunity [110]. The crystal structure of peptide-SLA-1*1502 (pSLA-1*1502) complexes with one peptide (nsp9-TMP9) (PDB code: 5YLX) provides the structural conformation basis of peptide presentation, and the overall structure is very similar to those of pSLA-1*0401 (PDB code: 3QQ3) and pSLA-3*hs0202 (PDB code: 5H94) [110,111,112]. The structural information displayed by the D pocket, as shown in Figure 2, plays a crucial part in determining and fixing the bound peptides, and provides a structural basis for designing effective peptide vaccines [111]. Study indicated that RdRp has the maximum number of conserved peptides by identifying SLA-1*1502-restricted potential epitopes peptides from whole genomes of different PRRSV strains; thus nsp9/10/11 (especially RdRp) may be the best targets for designing PRRSV vaccines to induce a CTL response to genetically heterologous strains [111]. Although the monomer structures of PRRSV nsp10 (PDB code: 6JDU) and nsp11 (PDB code: 5EYI) have been successfully solved, the structure and biological functional mechanisms of nsp9 remain elusive [95,98]. Table 1 shows the important protein structures and PDB codes that have been obtained in the PRRSV–host interaction network. Therefore, we are trying to explore the monomer or complex structure of nsp9, so as to provide a structural basis and new insight for treatments against PRRSV.

The review of the PRRSV–host interactions network helps us to understand the function of critical host factors involved in virus infection. More research is still needed to complete this complex network and provide the basis for the development of antiviral drugs and vaccines.

## 5. Conclusions

PRRSV is undoubtedly one of the most economically devastating swine pathogen, and has aroused sustained concern. In spite of constant efforts of the pathogenicity and immunology of PRRSV, it has always been a substantial challenge to design effective vaccines and drugs. MLV vaccines exist some safety issues. Compared with MLV vaccines, DIVA vaccines can distinguish PRRSV-specific antibodies produced by natural infection with WT viruses or by vaccination, and has become an important aim of many current research efforts. The acquisition of gene-edited pigs will be a potential antiviral strategy, but it’s a problem in acceptability of genetically modified foods. Structural information allows us to better understand the mechanisms of viral infection. A structure-based vaccine design approach may improve the antigenicity and create immunogens capable of eliciting robust neutralizing and protective immune responses.

The complex network of PRRSV–host interactions exists throughout the virus cycle. Only by studying the function of the PRRSV–host interactions in the overall process of PRRSV entry and infection, can we understand mechanisms of PRRSV replication and propagation, thereby providing the basis for antiviral strategies. Some host factors and viral proteins play important roles in the PRRSV–host network, but lack of structural information prevents us from understanding the mechanisms of infection thoroughly, Therefore, these host factors and viral proteins need to be explored to provide structural bases and new insights for treatments against PRRSV.

## Figures and Tables

**Table 1:** List of porcine reproductive and respiratory syndrome virus (PRRSV)–host interactions and their functions. Protein data bank (PDB) codes of structures are enclosed in the brackets.

Host	PRRSV	Function	Virus Genotypes	References
Heparan	M/GP5	Concentrate virions on the cell surface	PRRSV-1/PRRSV-2	[15]
pSn	M/GP5	PRRSV attachment and internalization receptor via clathrin-mediated endocytosis	PRRSV-1	[16]
CD163 (5JFB)	GP2/GP3/ GP4/GP5	Uncoating and genome release	PRRSV-1/PRRSV-2	[13,17]
CD151	3′ UTR RNA	Cooperate in infection	PRRSV-2	[18]
Simian vimentin	N (1P65)	Mediate transportation of the virus in the cytosol	PRRSV-2	[19]
CD209	GP5	Essential in PRRSV entry and infection	PRRSV-1/PRRSV-2	[20]
MYH9	GP5	Essential in PRRSV entry and infection	PRRSV-1/PRRSV-2	[21,22]
IFN-β	nsp1α (3IFU)	Suppress IFN by degrading CBP	PRRSV-2	[23,24]
	nsp1β (3MTV)	Suppress IFN by inhibiting both IRF-3 and NF-κB-dependent gene induction	PRRSV-2	[25]
	N	Suppress IFN by inhibiting the phosphorylation and nuclear translocation of IRF3	PRRSV-2	[26]
	nsp2	Suppress IFN by inhibiting the activation of the IRF-3 and NF-κB signaling	PRRSV-2	[27]
	nsp4 (5Y4L)	Suppress IFN by blocking NF-κB activation	PRRSV-2	[28]
	nsp11 (5EYI)	Suppress IFN by inhibiting the activation of the IRF-3 and NF-κB signaling, and inhibiting the formation and nuclear translocation of ISGF3 targeting IRF9	PRRSV-2	[29,30,31]
IL-1β	E	Increase the release of IL-1β	PRRSV-2	[32]
	nsp11	Inhibit the secretion of IL-1β	PRRSV-2	[33]
IL-10	N	Up-regulate IL-10 via NF-κB and p38 MAPK pathways in PAMs	PRRSV-2	[34]
	nsp1	Up-regulate IL-10	PRRSV-2	[35]
	Gp5	Up-regulate IL-10 through p38 MAPK and signal transducer and activator of transcription-3 (STAT3) activation	PRRSV-2	[36]
IL-17	nsp11	Induced IL-17 production depending on the PI3K-p38MAPK-C/EBPβ/CREB pathways	PRRSV-2	[37]
TRIM25	N	Competitively interact with TRIM25, thereby interfering with TRIM25-mediated RIG-I ubiquitination	PRRSV-2	[38]
TRIM22	N	Interact with TRIM22 thereby reducing virus replication	PRRSV-2	[39]
TRIM59	nsp11	Interact with TRIM59 thereby reducing virus replication	PRRSV-2	[40]
MiR-181	ORF4	Inhibit PRRSV replication	PRRSV-2	[41,42]
MiR-130	5′ UTR		PRRSV-2	[43]
MiR-23	ORF3		PRRSV-2	[44]
MiR-378	ORF7		PRRSV-2	[44]
MiR-505	ORF3/ORF5		PRRSV-2	[44]
PIAS1 SLA-I(5YLX)	nsp1α	Modulate degradation via SUMO E3 ligase activity	PRRSV-2 PRRSV-2	[45] [45,46]
Nup62	nsp1β	Inhibit host antiviral protein expression	PRRSV-1/PRRSV-2	[47]
STAT3	nsp5	Promote the degradation of STAT3 and interference with the JAK/STAT3 signaling	PRRSV-1/PRRSV-2	[48]
pRb	nsp9	Benefit the replication of PRRSV	PRRSV-2	[49]
NLRX1	nsp9	Restrict PRRSV replication	PRRSV-2	[50]
ZAP	nsp9	Repress PRRSV replication.	PRRSV-2	[51]
Fibrillarin Nucleolin Poly(A)-binding	N	Function remains to be further clarified	PRRSV-2	[52]

PRRSV: porcine reproductive and respiratory syndrome virus; UTR: untranslated region; ORF: open reading frame; N: nucleocapsid; IFN: interferon; CBP: cAMP response element-binding (CREB)-binding protein; NF-κB: nuclear factor-κB; IRF: IFN regulatory factor; ISGF3: IFN-stimulated gene factor 3; IL: interleukin; p38 MAPK: p38 mitogen-activated protein kinase; STAT3: signal transducer and activator of transcription-3; TRIM: tripartite motif; RIG-I: retinoic acid–inducible gene I; SUMO: small ubiquitin-related modifier; JAK: Janus kinase; PAMs: porcine alveolar macrophages; pSn: porcine sialoadhesin; MYH9: non-muscle myosin heavy chain 9; MiR: microRNA; PIAS1: protein inhibitor of activated STAT1; SLA-I: swine leukocyte antigen class I; Nup62: nucleoporin 62; pRb: retinoblastoma protein; NLRX1: nucleotide-binding oligomerization domain-like receptor X1; ZAP: zinc finger antiviral protein.

## Data Availability

Not applicable.
